# Bilateral implantation of a non-diffractive extended depth of focus intraocular lens in a pediatric patient with lamellar cataract

**DOI:** 10.1007/s10792-025-03728-7

**Published:** 2025-09-18

**Authors:** Sebastian Donald Marinow, Klemens Paul Kaiser, Jakob Wend, Thomas Kohnen

**Affiliations:** Department of Ophthalmology, Goethe-University, Theodor-Stern-Kai 7, 60590 Frankfurt Am Main, Germany

**Keywords:** Extended depth of focus, Cataract surgery, Infantile cataract, Refractive surgery

## Abstract

**Purpose:**

To present a case of a pediatric patient with infantile cataract, which was treated with lens extraction surgery and bilateral implantation of an extended depth of focus (EDOF) intraocular lens (IOL).

**Methods:**

A 13-year-old boy with bilateral lamellar cataract underwent standard phacoemulsification and bilateral implantation of a toric, non-diffractive EDOF IOL. Preoperative assessment included biometry, keratometry, and visual acuity testing. Postoperative follow-up included refraction, defocus curve, contrast sensitivity, and patient-reported outcomes.

**Results:**

After a follow-up of three months uncorrected and corrected distance visual acuity on both eyes was 0.1 logMAR. Uncorrected intermediate visual acuity (80 cm) was 0.2 logMAR (left eye) and 0.1 logMAR (right eye), while uncorrected near visual acuity (40 cm) was 0.5 logMAR (left eye) and 0.4 logMAR (right eye). The contrast sensitivity using Pelli Robson charts shows good results of 1.85 for both eyes. The patient indicated very low values concerning optical phenomena and was very satisfied. The patient reported that he occasionally uses reading glasses in close proximity.

**Conclusions:**

Bilateral implantation of a non-diffractive EDoF IOL resulted in high patient satisfaction with spectacle independence for distance and intermediate vision with an insignificant level of photic phenomena. The EDOF IOL appears to be a viable option for older pediatric patients undergoing cataract surgery.

## Background

Infantile cataracts are a common and important reason for lifelong visual impairment [1]. The prevalence of cataracts in childhood has been documented to range from 1 to 15 cases per 10,000 children, exhibiting significant regional variations [2, 3]. The etiology differs from the inheritance of an autosomal dominant trait or other genetic abnormalities to the point of metabolic causes such as diabetes, galactosemia, hypoglycemia or galactokinase deficiency [1]. Infantile Cataracts are an imminent danger for weak-sightedness by disrupting visual processing pathways of the central nervous system [4]. Thus, requiring prompt medical care in order to preserve lifelong vision potential and prevent vision loss, in particular amblyopia [4]. Cataracts are treated by surgical intervention through removal of the opacified lens and implantation of an artificial intraocular lens (IOL) [4, 5].

Lamellar cataract, a form of infantile cataract, is characterized by the impairment of the lamellae surrounding the fetal nucleus, typically manifesting subsequent to fixation. This condition is predominantly bilateral and progressive in nature, frequently necessitating surgical intervention with implantation of an IOL prior to the onset of school age. However, it is noteworthy that this form of cataract can also persist as a subclinical condition for an extended duration [6].

In recent years, IOL with greater range of depth of field have been utilized to provide pseudo-accommodation and achieve enhanced stereopsis in pediatric patients. Extended depth of focus (EDOF)- IOLs are designed to improve visual acuity across a continuous range, particularly in the intermediate zone, by extending the depth of focus. Non-diffractive EDOF lenses achieve this through wavefront shaping, offering advantages such as fewer photic phenomena and better contrast sensitivity compared to diffractive multifocal IOLs [7–9]. However, challenges have been associated with this approach, including the calculation of IOL power, surgical complications, and the need for repeat surgery. Further disparities that must be taken into account pertain to anatomical and physiological characteristics in children (e.g. larger pupil diameter, increased healing response, and incomplete eye growth) [10]. These challenges differ from those experienced by adult cataract patients [10, 11].

In this case report, we present a child with bilateral lamellar cataract and subsequent implantation of a non-diffractive EDOF-IOL.

## Case presentation

**Fig. 1:**
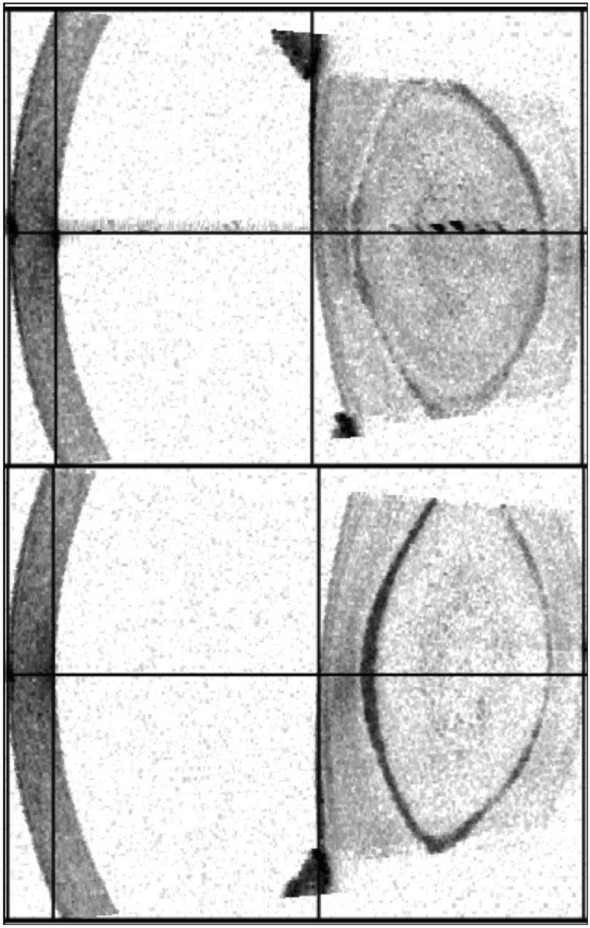
The optical coherence tomography (OCT) image of the IOLMaster 700 (Carl Zeiss Meditec AG, Jena, Germany) reveals the presence of lamellar cataracts in both eyes of the patient

A 13-year-old male patient presented to the Department of Ophthalmology, University Hospital Frankfurt, Germany, with an infantile cataract on both eyes. In our examination, we found a bilateral lamellar cataract and an impaired visual acuity in both eyes (Fig. 1).

Although cataract extraction had been recommended before the age of six, the parents opted against early surgery. As a result, amblyopia therapy through occlusion was initiated with a delay, once visual acuity had deteriorated and surgical intervention was still not desired. Occlusion therapy was initiated at the age of six for a period of two years for the prevention of amblyopia.

The patient reports experiencing difficulty reading from the blackboard, even with his glasses. His corrected distance visual acuity (CDVA) was 0.3 logarithm of the minimal angle of resolution (logMAR) on the right eye and 0.5 (logMAR) on the left eye. Manifest spherical refractive error was + 3.50 diopters (D) with an astigmatism of -3.25 D at 177 degrees (°) and + 1.75 D and − 2.50 D at 11° on the right and left eye, respectively.

The endothelial cell count was 2476 cells/mm^2^ in the right eye and 2860 cells/mm^2^ in the left eye. The axial length was 22.51 mm and 23.17 mm in the right and left eye, respectively. The flat keratometry was 43.06 D and 42.94 D in the right and left eye, respectively, and the steep keratometry was 46.22 D and 45.60 D in the right and left eye, respectively. Corneal tomography showed a symmetric bow-tie with corneal astigmatism measuring -3.16 D at 172° in the right and -2.66 D at 4° in the left eye.

The patient and his parents had already been thoroughly informed and explicitly expressed the desire to achieve independence from glasses for the majority of activities following cataract surgery. Following careful deliberation regarding the patient’s preferences and a thorough evaluation of the merits and drawbacks inherent to each IOL model, the non-diffractive toric EDOF IOL was ultimately deemed the optimal choice.

With the consent of the parents and the patient, a bilateral standard cataract operation was performed under general anesthesia. The corneal incisions were carried out by one 2.2-mm main temporal incision and two paracentesis. The phacoemulsification was conducted using standard ultrasound technique (Alcon, Centurion Vision System). The implanted toric EDOF IOL (AcrySof IQ Vivity IOL DFT615, Alcon, Forth Worth, TX, USA) had a power of + 23.00 D and a torus of 3.75 D on IOL plane. In order to stabilize the IOL position, particular attention was paid to ensuring that the capsulorhexis size was sufficient (roughly 4.8 mm) and that the IOL optic center was properly centered within the capsular bag. Toric alignment was performed with preoperative marking in the sitting position. The IOL power was calculated with the Barrett Toric Calculator, which is based on biometric data from the IOLMaster 700 (Carl Zeiss Meditec AG).

**Fig. 2:**
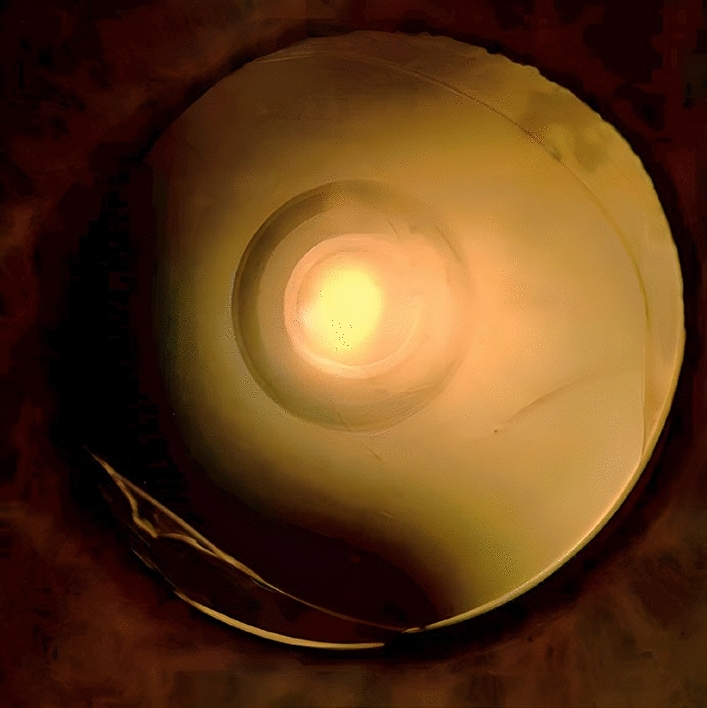
The postoperative slitlamp photograph illustrates the intraocular lens positioned within the capsular bag. The central wavefront-shaping ring is clearly visible

**Fig. 3:**
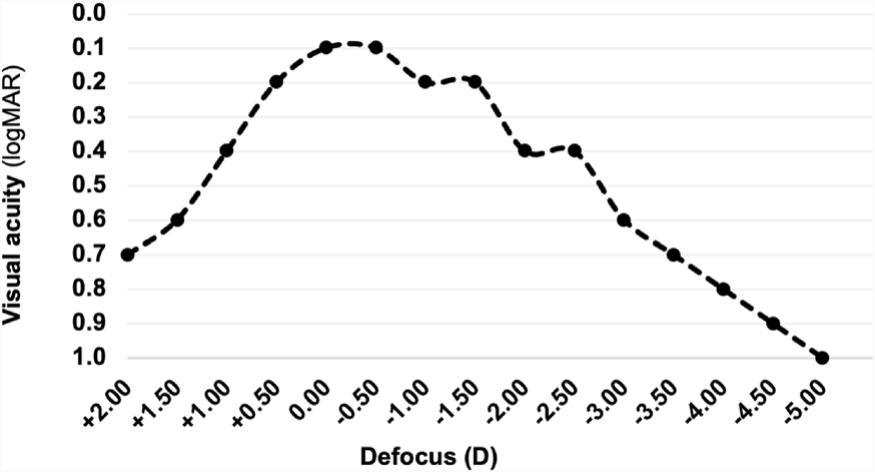
The patient’s binocular defocus curve indicates a range of 0.1 to 0.2 logMAR between + 0.50 to − 1.50 diopters (D), accompanied by a subsequent decline in defocus

**Fig. 4:**
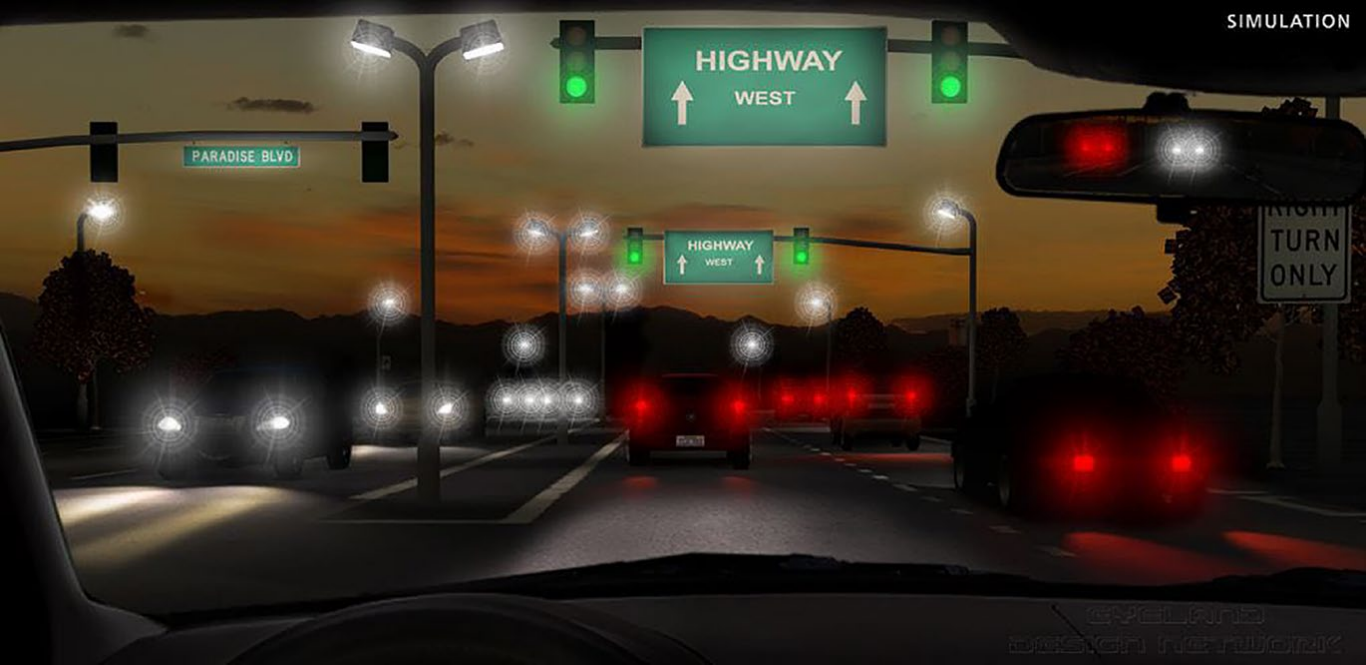
The subjective optical phenomena experienced by the patient three months after cataract surgery were reproduced using the halo and glare simulator (Eyleand-Design Network GmbH, Vreden, Germany)

Three months after the cataract surgery the patient underwent a follow-up visit with a detailed ophthalmologic examination. Figure 2 shows the patient’s right eye with the IOL placed in the capsular bag. The binocular defocus curve of the patient is shown in Fig. 3. Manifest refractive spherical error was + 0.50 D on the right and + 0.25 D on the left eye. Uncorrected distance visual acuity (UDVA) and CDVA on both eyes was 0.1 logMAR. Uncorrected intermediate visual acuity (UIVA) at 80 cm was 0.2 logMAR on the right eye and 0.1 logMAR on the left eye, and uncorrected near visual acuity (UNVA) at 40 cm was 0.5 logMAR on the right eye and 0.4 logMAR on the left eye. The contrast sensitivity measured using the Pelli-Robson chart shows excellent results of 1.85 decimal value for both eyes. Photic phenomena were evaluated using a halo and glare simulator (Eyleand-Design Network GmbH, Vreden, Germany). The patient was asked to plot optical phenomena on a scale between 0 and 100 (0 lowest value, 100 highest value) during bright light, intermediate light and weak light in several categories: These categories included halos, starbursts, fold-overs as well as blurred-images. These values were reproduced into the above-mentioned halo and glare simulator by Eyleand-Design Network GmbH, Vreden, Germany and show the following image in Fig. 4. The patient expressed overall satisfaction with the procedure. He reported achieving spectacle independence for the majority of his daily activities at intermediate and long distances, with only a reliance on spectacles for near vision. He stated that he would recommend the same intraocular lens to other patients and undergo the same procedure again.

## Discussion

As well as any other medical care, treating infantile cataract has the objective of enhancing the visual function [2]. It therefore requires individual decision-making on the time of surgical intervention: It depends on the severity of the lens opacity as well as the age of the patient at time of presentation [2]. Although surgical intervention is often the only and best therapy to restore visual function it is important to consider that there is a higher risk of intraoperative complications [2]. This is due to the differences of the pediatrics’ ocular anatomy and physiology: Pediatric patients are known to have a more elastic anterior capsule, a more pliable sclera as well as a higher vitreous pressure compared to adults, thus leading to more intraoperative complications [2]. We therefore advise that the decision on surgical intervention for treating infantile cataracts requires an even more careful and broad evaluation when treating younger patients. In addition, these interventions should only be carried out by experienced surgeons due to the higher risk of complications.

Not only do the anatomy and physiology of paediatric eyes differ from those of adults’ eyes. Pediatric eyes face rapid growth with a change in the axial length and corneal diameter [4, 12]. Due to continuous growth of the pediatric eye, IOL selection can be challenging. This pediatric patient reported spectacle independence for the far and intermediate range and being in need for reading glasses for the near range. A refractive intervention has the ultimate goal of spectacle independence. A correction for the near range for pediatric patients is hardly accomplishable long-term: The increase of the axial length of his pediatric eyes will result in a stronger refraction power, thus leading to a change of focus in leading to myopia [4]. In order to keep his growing eyes in focus, an optical correction with spectacles would be necessary again [4]. IOL calculation for pediatric patients` is therefore often balanced to a small hyperopic error in the pseudophakic eye when the pediatric patient reaches adulthood and avoiding extreme myopia in the pseudophakic eye to circumvent an IOL exchange later in life [4, 13].

Given the high incidence of secondary cataracts in children, primary posterior capsulotomy combined with anterior vitrectomy is frequently performed [14]. In this particular case, however, the procedure was deliberately omitted due to the patient’s age of 13 years, as a subsequent YAG laser capsulotomy was considered a sufficiently viable alternative. Furthermore, careful consideration must be given to whether the posterior capsule should be opened, as studies have shown that this may complicate IOL exchange [15]. This is also relevant in pediatric patients, as in the event of implantation of a three-piece IOL in the ciliary sulcus, no enhanced optic would be available [15].

In the United States IOL implantation in pediatric patients is even considered “off label “ because IOLs were not tested in children during their FDA market approval process [4]. The accuracy of IOL calculation formulas decreases with shorter axial lengths and younger patient age, and is particularly unreliable during the first five years of life [4, 16]. To prevent refractive shifts as axial elongation progresses, the IOL was calculated for mild hyperopia. The EDOF IOL offers tolerance to refractive changes, making it particularly suitable for this age group.

However, the patient in this case report is a teenager whose IOL calculation is more predictable than in an infant patient, with a reduced likelihood of substantial ocular dimension change compared to an infant patient. Consequently, this constitutes a substantial distinction when compared to a congenital cataract, which necessitates lens removal during the initial weeks of life. The implantation of a toric presbyopia-correcting IOL in an infant is not recommended due to the unpredictable growth of the bulb and consecutive lack of benefit.

In pediatric cataract surgery, IOL selection is challenging due to the developmental state of the visual system and the risk of amblyopia [17]. Monofocal IOL remain the standard but offer limited functional vision. Multifocal IOL are generally avoided in children due to reduced contrast sensitivity and increased photic phenomena [17]. EDOF IOL, by contrast, provide an extended range of focus with fewer optical side effects and better contrast performance, making them a potential alternative in selected pediatric cases. In our patient, the EDOF IOL was well tolerated and supported visual rehabilitation without compromising image quality. While pediatric data remain limited, this case suggests that EDOF IOL may offer a functional and safe option in appropriately selected adolescents.

In children and adolescents with amblyopia secondary to congenital cataracts, the use of EDOF IOLs raises questions about their influence on postoperative amblyopia therapy and visual rehabilitation. EDOF IOLs are designed to provide an extended range of focus, potentially reducing the dependence on spectacles and improving contrast sensitivity, which may facilitate visual training [18]. In this case, the patient had already undergone occlusion therapy earlier in childhood, and visual function was considered trainable. Postoperatively, the EDOF IOL provided satisfactory image quality across different focal ranges, supporting ongoing visual development. While long-term data on EDOF IOLs in pediatric populations remain limited, the positive functional outcome in this case suggests that, with appropriate patient selection and prior amblyopia treatment, EDOF IOLs may not hinder—and may even support—visual rehabilitation. Our follow-up exam after three months showed good functional results and high patient satisfaction after bilateral EDOF IOL implantation. However, this short follow-up period of three months does not allow conclusions on long-term results [7].

In this case, the AcrySof IQ Vivity IOL was selected due to its non-diffractive wavefront-modifying technology, which offers enhanced depth of field with minimal occurrence of phenomena such as halos or glare. This represents a substantial advantage over diffractive EDOF-IOLs or trifocal lenses, particularly in pediatric patients who may experience residual amblyopia. Trifocal IOLs can also provide good results for far, near and intermediate distances and should therefore also be considered as a feasible alternative to EDOF IOLs [19]. Nonetheless, the inability to preoperatively exclude amblyopia rendered implantation of a trifocal IOL in the adolescent patient inadvisable. Moreover, the patient expressed a preference for wearing reading glasses rather than accepting the potential optical phenomena associated with the trifocal diffractive IOL. An additional advantage of the implantation of EDOF IOLs in juvenile patients is that the EDOF IOLs adapt better to a myopic shift due to their defocus curve. In this connection, it is important to differ between non-diffractive and diffractive IOLs: This implanted EDOF IOL is a non-diffractive wavefront-shaping IOL which has less optical phenomena than a diffractive IOL [8, 20]. In general, trifocal IOLs are known to show better results for near visual acuity while EDOF IOLs show better results for the intermediate range [9]. Studies show that the majority of trifocal IOLs offer more spectacle independence but have the inconvenience of disturbing optical phenomena while EDOF IOLs offer a better quality of vision but are inferior in visual acuity in the near range to trifocal IOLs [9]. We gave great importance to avoiding optical phenomena through intraocular lens implantation on pediatric eyes and therefore decided to desist from multifocal IOLs for a pediatric patient in favor of an EDOF IOL. Studies with a longer follow-up period are needed to be performed on visual outcomes after EDOF IOL implantation in pediatric eyes undergoing cataract surgery.

## Conclusion

In our case, after the lens extraction of a lamellar cataract and implantation of a toric EDOF IOL, we achieved spectacle independence for the distance and intermediate range. As a consequence of this, the patient is not reliant on varifocals. In the event that a patient exhibits a stable and consistent refraction power, the implantation of an IOL with an extended range of depth of field could serve as a viable alternative for patients diagnosed with pediatric cataracts, with the objective of enhancing their spectacle independence. In this case, the lens has good results for visual acuity and insignificant levels of optical phenomena. Bilateral implantation of this toric non-diffractive EDOF IOL was tolerated well with high patient satisfaction.

## Data Availability

No datasets were generated or analysed during the current study.

## Abbreviations

CDVA: Corrected distance visual acuity
EDoF: Extended depth of focus
IOL: Artificial intraocular lens
logMAR: Logarithm of the minimal angle of resolution
OCT: Optical coherence tomography
RoF: Range of depth of field
UDVA: Uncorrected distance visual acuity
UIVA: Uncorrected intermediate visual acuity
UNVA: Uncorrected near visual acuity
